# Probing the Solution Structure of IκB Kinase (IKK) Subunit γ and Its Interaction with Kaposi Sarcoma-associated Herpes Virus Flice-interacting Protein and IKK Subunit β by EPR Spectroscopy*[Fn FN2]

**DOI:** 10.1074/jbc.M114.622928

**Published:** 2015-05-14

**Authors:** Claire Bagnéris, Kacper B. Rogala, Mehdi Baratchian, Vlad Zamfir, Micha B. A. Kunze, Selina Dagless, Katharina F. Pirker, Mary K. Collins, Benjamin A. Hall, Tracey E. Barrett, Christopher W. M. Kay

**Affiliations:** From the ‡ Department of Biological Sciences, Institute of Structural and Molecular Biology, Birkbeck College, University of London, London WC1E 7HX, United Kingdom,; § Institute of Structural and Molecular Biology, Darwin Building, University College London, Gower Street, London WC1E 6BT, United Kingdom,; ¶ MRC Centre for Medical Molecular Virology, UCL Cancer Institute and National Institute for Biological Standards and Control, Blanche Lane, South Mimms, Potters Bar, Herts EN6 3QG, United Kingdom,; ‖ MRC Cancer Unit, Hutchison/MRC Research Centre, Cambridge Biomedical Campus, University of Cambridge, Cambridge CB2 0XZ, United Kingdom, and; ** London Centre for Nanotechnology, University College London, 17-19 Gordon Street, London WC1H 0AH, United Kingdom

**Keywords:** electron paramagnetic resonance (EPR), molecular dynamics, molecular modeling, NF-kappa B (NF-κB), protein structure, virology, IKKγ, NEMO, signalosome, vFLIP

## Abstract

Viral flice-interacting protein (vFLIP), encoded by the oncogenic Kaposi sarcoma-associated herpes virus (KSHV), constitutively activates the canonical nuclear factor κ-light-chain-enhancer of activated B cells (NF-κB) pathway. This is achieved through subversion of the IκB kinase (IKK) complex (or signalosome), which involves a physical interaction between vFLIP and the modulatory subunit IKKγ. Although this interaction has been examined both *in vivo* and *in vitro*, the mechanism by which vFLIP activates the kinase remains to be determined. Because IKKγ functions as a scaffold, recruiting both vFLIP and the IKKα/β subunits, it has been proposed that binding of vFLIP could trigger a structural rearrangement in IKKγ conducive to activation. To investigate this hypothesis we engineered a series of mutants along the length of the IKKγ molecule that could be individually modified with nitroxide spin labels. Subsequent distance measurements using electron paramagnetic resonance spectroscopy combined with molecular modeling and molecular dynamics simulations revealed that IKKγ is a parallel coiled-coil whose response to binding of vFLIP or IKKβ is localized twisting/stiffening and not large-scale rearrangements. The coiled-coil comprises N- and C-terminal regions with distinct registers accommodated by a twist: this structural motif is exploited by vFLIP, allowing it to bind and subsequently activate the NF-κB pathway. *In vivo* assays confirm that NF-κB activation by vFLIP only requires the N-terminal region up to the transition between the registers, which is located directly C-terminal of the vFLIP binding site.

## Teaser

**Background:** The IκB kinase subunit γ (IKKγ) is an essential modulator of the IKK signalosome whose solution structure is unsolved.

**Results:** IKKγ is a parallel coiled-coil comprising two registers accommodated by a twist.

**Conclusion:** The response of IKKγ to binding of signaling partners is twisting and stiffening, rather than major rearrangement.

**Significance:** Viral activation of IKKγ exploits the structural motif of the twist.

## Introduction

Activation of the canonical nuclear factor kappa-light-chain-enhancer of activated B cells (NF-κB)^7^ transcriptional pathway occurs in response to a wide variety of cellular stimuli, including cell differentiation, infection, and stress responses (1). It is normally tightly regulated since constitutive activation can lead to prolonged cellular survival and the production of inflammatory cytokines both of which have been directly linked to cancer and inflammatory diseases. The pathway converges on a set of NF-κB transcription factors that in resting cells are localized to the cytoplasm because of their association with inhibitory IκB proteins (2–4). Liberation of the NF-κB transcription factors and their transition to the nucleus requires degradation of the IκBs that are first phosphorylated and subsequently targeted for proteolysis by the 26S proteasome following Lys-48 ubiquitination. Phosphorylation is facilitated by IκB kinase (IKK) or signalosome that minimally comprises the kinase subunits IKKα and/or IKKβ together with a modulatory element IKKγ (also known as NEMO: NF-κB essential modulator). This assembly is the target for several viruses since its constitutive activation results in the downstream overproduction of proteins that indirectly promote viral propagation and proliferation through their capacity to prolong cellular survival (5, 6). One such virus is Kaposi sarcoma-associated herpes virus (KSHV) that during its latent phase encodes vFLIP (7). The pathogenicity of vFLIP appears to derive from its capacity to render IKK constitutively active by associating with its modulatory element IKKγ (8, 9). This prolonged activation has been directly linked to Kaposi sarcoma (KS) and other KSHV associated malignancies that includes primary effusion lymphoma (PELs) and multicentric Castleman disease where knockdown of vFLIP alone is sufficient to arrest the growth of KS tumors and kill PELs cells (10, 11). The crystal structure of the vFLIP·IKKγ complex (12) has revealed the nature of this interaction at the atomic level, which was later verified *in vivo* (13). However, the mechanism by which vFLIP is able to activate the IKKα and IKKβ kinases remained unclear, especially because they associate with the N terminus of IKKγ, which is more than 200 residues from the vFLIP binding site. Furthermore, recent studies have shown that the ability of vFLIP to activate the canonical NF-κB pathway appears to be independent of up and downstream effectors such as: tumor necrosis factor (TNF) receptor-associated factors (TRAF)-2, TRAF-3, TRAF-6; linear ubiquitin chain assembly complex (LUBAC); and transforming growth factor (TGF)-β-activated kinase 1 (TAK1) that are all essential for the mechanisms utilized by pro-inflammatory cytokines (14).

It has been proposed that vFLIP activation of IKK involves conformational changes within the IKKγ molecule that effectively switches the assembly from an inactive to an active state that would favorably juxtapose IKKα/β for either trans or autophosphorylation (12). Because there is currently neither a crystal structure of full-length IKKγ nor a fragment that encompasses both the kinase and vFLIP binding sites (either alone or in the relevant complexes) to allow direct testing of this hypothesis in terms of both local and global transitions, we used electron paramagnetic resonance (EPR) spectroscopy to determine distances between spin-labeled cysteine residues introduced at intervals along IKKγ. These allowed *in silico* models of IKKγ to be validated, enabling a solution structure of IKKγ to be obtained for the first time. Measurements were also performed in the presence of vFLIP and separately an IKKβ fragment comprising the IKKγ binding site to provide readout of any induced conformational changes.

## Experimental Procedures

### 

#### 

##### Cloning

Native full-length human IKKγ degrades at the N and C termini prompting the use of a truncated construct, encompassing residues 40–354. Human IKKγ (40–354) was amplified by polymerase chain reaction (PCR) using the plasmid pGEX-KT IKKγ (8). This was performed using the primers 5′-TTTTGGATCCCACCTGCCTTCAG-3′ and 5′-TTTTGAATTCCTAGTCCTCGATCCTGGC-3′ containing the BamHI and EcoRI (underlined) restriction sites, respectively. The resulting PCR product was cloned into pETM442 vector (details can be found in the supplemental data of Ref. 12) that had previously been digested using the same enzymes. The resulting construct included a tobacco etch virus (TEV) protease cleavable N-terminal 6His-NusA tag directly 5′ to the IKKγ insert as an aid to protein solubilization and purification. To prevent further proteolysis, Gln-83 was mutated to alanine. All IKKγ mutants were produced using the QuikChange kit (Agilent). A list of primers is provided (Supplemental Table S1). The IKKγ interaction domain of IKKβ (amino acids 644 to 756) was PCR amplified from the pRC-actin IKKβ plasmid that incorporated the full human construct using the primers 5′-CACCGTCCGGCTGCAGGAG-3′ and 5′-TCATGAGGCCTGCTCCAGGC-3′. It was cloned into pET151/d-TOPO® (Invitrogen) to provide an N-terminal 6His-tag cleavable by TEV protease. pETM6T1 vFLIP (1–178) from KSHV, also encompassing a TEV protease cleavable N-terminal 6His-NusA-tag, is described elsewhere (12).

##### Expression

Wild type and mutant pETM442 IKKγ (40–354) and pETM6T1-vFLIP (1–178) were transformed into BL21(DE3)Star (Invitrogen) that harbored the pRARE2 plasmid encoding rare tRNAs (Novagen). pET151 IKKβ (644–756) was transformed into Rosetta-gami B (DE3) (Novagen). The cells were grown in 100 ml of 2YT medium (1.6% (w/v) bacto-tryptone (Invitrogen), 1% bacto-yeast extract (Invitrogen) and 0.5% NaCl, adjusted to pH 7.2) containing chloramphenicol (35 μg ml^−1^) and supplemented with either ampicillin (100 μg ml^−1^) for IKKγ, kanamycin (24 μg ml^−1^) for vFLIP, or kanamycin (24 μg ml^−1^), ampicillin (100 μg ml^−1^) and tetracyclin (12.5 μg ml^−1^) for IKKβ, and grown at 37 °C overnight. 1 in 100 dilutions of overnight culture were inoculated into 2YT medium and grown to an OD_600_ of 1.0 at 37 °C before induction with 1 mm isopropyl 1-thio-β-d-galactopyranoside (IPTG) for IKKγ and vFLIP or 0.1 mm IPTG for IKKβ, and allowed to grow at: 30 °C for 3 h (IKKγ); 16 °C overnight (vFLIP); or 20 °C overnight (IKKβ). The cells were then harvested by centrifugation, washed with buffer A (200 mm NaCl, 25 mm Tris, pH 8.5) and stored at −70 °C.

##### Protein Purification

Cell extracts were re-suspended in buffer A supplemented with DNase I (10 μg ml^−1^ final concentration) and EDTA-free protease inhibitor mixture tablets (Roche). After sonication on ice, lysates were clarified by centrifugation (46,000 × *g* for 1 h at 4 °C) and supernatants filtered through a 0.45 μm filter prior to loading onto a 5 ml of HisTrap^TM^ FF column (GE-Healthcare). The column was washed with 20 column volumes (CVs) of buffer A containing 50 mm imidazole and the protein eluted with 5 CVs of buffer A containing 500 mm imidazole. The tags were cleaved with TEV protease and overnight dialysis against buffer A supplemented with 1 mm DTT and 0.5 mm EDTA for vFLIP and IKKβ. For IKKγ, the buffer pH was adjusted to 7.5 and maintained at this pH for the following anion exchange step. Cleaved protein solutions were diluted to 50 mm NaCl before loading onto a 5 ml HiTrap Q FF column (GE-Healthcare) and the proteins eluted with a 50 to 500 mm NaCl linear gradient (20 CVs). IKKγ was then spin-labeled as described in the following section. IKKβ and vFLIP were dialyzed against 25 mm Tris, pH 7.5, 200 mm NaCl, 10% glycerol, and concentrated to 2 mg ml^−1^ prior to addition of spin-labeled IKKγ.

##### Spin Labeling of IKKγ and Purification of Protein Complexes

**FIGURE 1.**
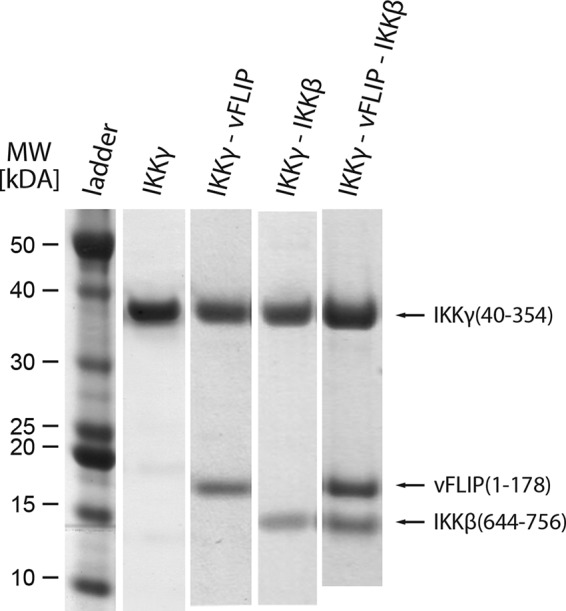
**SDS-PAGE Coomassie-stained gels of the final size-exclusion profiles obtained for: IKKγ; IKKγ·vFLIP complex; IKKγ·IKKβ complex; IKKγ·vFLIP·IKKβ ternary complex of the 56–95 double mutant.**

IKKγ was incubated with a 20-fold excess of 3-(2-iodoacetamido)-proxyl (Aldrich) spin label overnight at 4 °C in the dark. The sample was concentrated to less than 1 ml using a 30 kDa cut-off Vivaspin centrifugal concentrator (Vivascience) before removal of free spin label by size exclusion chromatography using a Superdex 200 HR 10/60 column, pre-equilibrated with 25 mm Tris, pH 7.5, 200 mm NaCl, 10% glycerol. Prior to spectroscopic analysis, the IKKγ buffer was exchanged through concentration and dilution into one in which H_2_O was replaced by D_2_O. Alternatively, IKKγ was incubated with either IKKβ or vFLIP at 4 °C overnight for complex formation. These were then purified by size exclusion chromatography as described for IKKγ alone SDS-PAGE Coomassie-stained of final size-exclusion profiles following purification for the 56–95 double mutant are depicted in Fig. 1. All other constructs gave similar results. The complexes were subsequently buffer exchanged into D_2_O buffer before analysis. The stability of each labeled mutant was assessed by comparing its elution volume on a size exclusion chromatography to that of wild type IKKγ: all had similar profiles. The final concentration of samples for spectroscopic analysis was in the 25–100 μm range. These gave labeling efficiencies of above 70% as determined by continuous-wave EPR.

##### EPR Spectroscopy

EPR-based distance measurements were performed at 50 K after flash freezing the samples in liquid nitrogen on an ELEXSYS E580 spectrometer (Bruker) operating at 9.6 GHz equipped with an ER 4118 X-MD5 resonator, a CF935 continuous flow cryostat (Oxford Instruments) and ITC503 temperature controller. The 4-pulse Double Electron-Electron Resonance sequence (15) used was π/2(ν*_obs_*)-τ_l_-π(ν*_obs_*)-*t*-π(ν*_pump_*)-(τ_l_+τ_2_-*t*)-π(ν*_obs_*)-τ*_2_-echo*, where the observer pulse length was 16 ns for π/2 and 32 ns for π pulses. The pump pulse length was 12 ns. The long interpulse delay (τ*_2_*) was 3500–4500 ns for the single mutants and 4000–5500 ns for the double mutants. All other parameters were according to Ref. 15 with τ_1_ = 400 ns and Δτ_1_ = 56 ns. Data points were collected in 8 ns time steps. The total measurement time for each sample was in the range of 8 to 36 h. The spectra were analyzed using the program DeerAnalysis2011 (16). The background was corrected by a homogeneous three-dimensional fit and the distance distributions evaluated by Tikhonov regularization.

**FIGURE 2.**
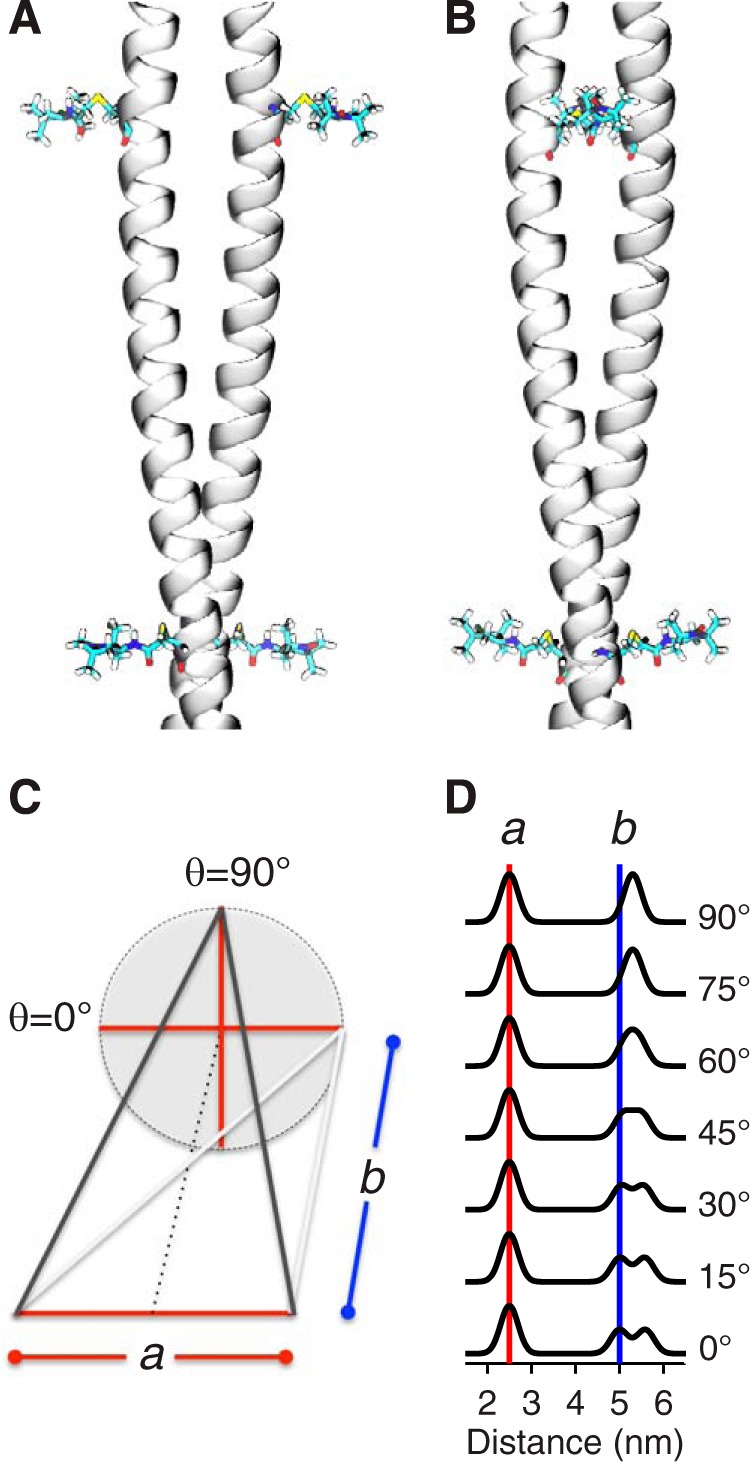
**Diagram illustrating how the register of the coiled-coil translates into distance distributions between two pairs of nitroxide spin labels.** Limiting cases in which (*A*) all 4 labels are in a plane or (*B*) the two vectors joining the pairs are perpendicular. *C*, geometry is constrained by the intra-pair distance (designated *a*), the inter-pair distance (designated *b*) and dihedral angle (designated θ) between the vectors joining the pairs. *D*, illustration of the variation of the distance distribution for two pairs of labels as a function of θ for *a* = 2.5 nm and *b* = 5 nm with Gaussian linewidth of 0.2 nm.

Pairs of residues were chosen so that the intra-pair distance was 2–3 nm, and the inter-pair distance was 4–6 nm. This choice ensured that the r^−3^ dependence of the dipolar interaction, whereby doubling the distance results in 8-fold lower dipolar frequency, separated the inter-pair from the intra-pair frequency while keeping the longer distance accessible. As illustrated in Fig. 2, in a coiled-coil, the geometry is constrained by the intra-pair distance (designated *a*), and the inter-pair distance (designated *b*) and dihedral angle (designated θ) between the vectors joining the pairs. There are two long distances between the labels, which are given by the expression: √*a*^2^/2(1 + cosθ) + *b*^2^. The two limiting cases are when the vectors joining the pairs are parallel (Fig. 2*A*) or when the vectors joining the pairs are perpendicular (Fig. 2*B*). In the former θ = 0°, and the labels form a rectangle with two distinct long distances: corresponding to the sides, *b,* and the diagonals √*a*^2^ + *b*^2^ (Fig. 2*C*, *gray lines*). In the latter θ = 90° and there is a single long distance: √*a*^2^/2 + *b*^2^ (Fig. 2*C*, *black lines*). As illustrated in Fig. 2*D*, the shape of the distance distribution is strongly dependent on θ, and hence the geometry of the coiled-coil and changes thereto may be evaluated by EPR spectroscopy.

Note that for several cases, fewer spin pairs were observed for the IKKγ-IKKβ or IKKγ-vFLIP complexes; this is apparent from the relative modulation depth of the dipolar evolution. However, such differences can arise from the labeling efficiency varying between the samples, making interpretation ambiguous so we do not pursue it here.

##### MD Simulations

Coarse-grained MD simulations were performed using the MARTINI force field over 1 μs (17, 18). In brief, ∼4 non-hydrogen atoms are modeled as a single coarse-grained particle. Non-bonded Lennard-Jones interactions are based on 4 classes of particles (polar, apolar, non-polar, and charged), which are further divided into up to 5 subtypes. Interactions between different classes and sub-classes of particle are calculated based on an interaction table with 9 distinct interaction types. Furthermore, Lennard-Jones interactions were shifted to zero between 0.9 and 1.2 nm. Electrostatics were treated coulombically with a cut off at 1.2 nm (interactions are shifted to zero between 0 and 1.2 nm). α-helical secondary structure was maintained through dihedral angle restraints. Temperature and pressure were coupled at 323 K and 1 bar, respectively using the Berendsen weak coupling algorithm (T_T_ = 1 ps and T_P_ = 10 ps). Simulations were performed with Gromacs 4.5 (19). This generated an extended simulation trajectory from which 2,500 frames were used as template structures in MODELLER to generate new all-atom models of IKKγ. As each of these structures represents a distinct point in the dynamic ensemble, each of the structures has similar limitations to the original model. To include all of the conformational variability observed across the simulation, distance predictions using a rotamer library approach (20) were made for the labels in each frame and combined to generate a single distribution representing all label positions for all observed backbone bending, across the complete dynamic ensemble. This approach is a variation on serial multiscale modeling approaches (21), which explicitly samples across the entire trajectory rather than the end point alone.

##### NF-κB Luciferase Reporter Assays

Reporter assays were performed on 70Z/3 or Jurkat IKKγ-null cells stably reconstituted with IKKγ mutants as previously described (13). Reconstituted cells were transduced with a lentiviral vector (LV) encoding an NF-κB responsive luciferase reporter. 5 × 10^4^ cells were seeded in optical bottom 96-well plate and transduced with a vFLIP encoding LV. 48 h later, 50 μl of BrightGlo luciferase substrate (Promega) was added to each well and the NF-κB-induced luminescence was detected using Varioskan Flash multimode reader (Thermo Scientific). Where indicated cells were treated for 6 h with lipopolysaccharide (10 μg ml^−1^) prior to substrate addition.

## Results

### 

#### 

##### Analysis of IKKγ using Single Pairs of Spin Labels

**FIGURE 3.**
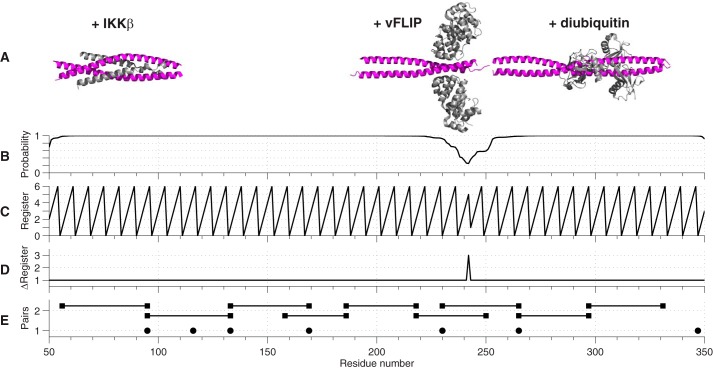
*A*, crystal structures of fragments of IKKγ (*magenta*) in complex with IKKβ, vFLIP, and diubiuitin (*gray*) aligned according to their position in the sequence. *B*, probability of a region adopting a coiled-coil arrangement based on a window of 28 residues predicted from the primary sequence of IKKγ. *C*, predicted position of residues within a coiled-coil based on the heptameric repeat of the motif: each residue has a predicted position from 0–6 and a regular zigzag pattern implies a perfect coiled-coil. *D*, increment of the register of a residue with respect to its predecessor. An increment of +1 implies a perfect coiled-coil. *E*, positions of nitroxide spin labels for singly labeled IKKγ (*solid circles*) and doubly labeled IKKγ (*solid squares* joined by *solid lines*).

The existing crystal structures of IKKγ fragments (12, 22–25) all exhibit coiled-coil structures (Fig. 3*A*). Furthermore, 4 different coils prediction algorithms (26–29) suggest that the entire molecule (excluding the C-terminal zinc finger) adopts a parallel coiled-coil conformation concomitant with a switch in helical register close to Val-250. The results from Multicoil2 (28) are depicted in Fig. 3, *B–D*. To validate the prediction we employed EPR distance measurements; nitroxide spin-labeling of a single cysteine in a homo-dimeric protein such as IKKγ automatically gives rise to a pair of labels, whose separation may be measured by EPR spectroscopy.

**FIGURE 4.**
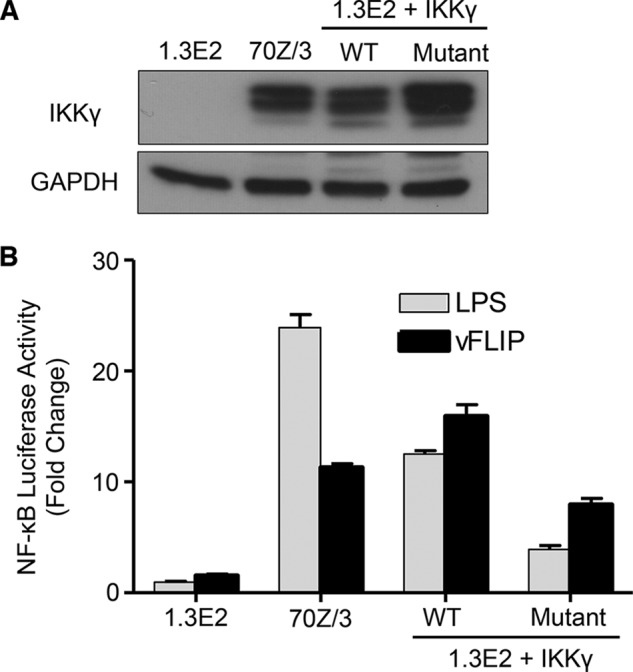
**NF-κB reporter assays for the vFLIP mutant in which all 5 out of 6 cysteines were mutated to serine.** *A*, Western blot analysis of IKKγ expression level in IKKγ knock-out mouse PreB cell line 1.3E2, respective parental cells 70Z/3 and IKKγ wild type or mutant reconstituted 1.3E2 cells. *B*, NF-κB activation was measured in the above-mentioned cell lines using the BrightGlo Luciferase assay system, 6 h after stimulation with LPS (10 μg ml^−1^) or 48 h after transduction with KSHV vFLIP LV (MOI = 50). Bars represent mean fold induction values ± S.D.

IKKγ, however, contains 6 native cysteine residues distributed throughout the sequence. To establish whether their removal (in order to produce mutants containing a single cysteine for spin-labeling studies) would have a deleterious effect on the activity of IKKγ, a mutant involving the full-length protein in which 5 cysteines were mutated to serines (leaving only Cys-167) was constructed and tested *in vivo* using a NF-κB luciferase reporter assay. Although the levels of NF-κB activation were reduced relative to wild type IKKγ, the mutant was still able to activate the pathway when stimulated by vFLIP (Fig. 4). In contrast, significant attenuation for this mutant was observed following stimulation using lipopolysaccharide (LPS) indicating that at least some of these residues have a role in cytokine induced activation. Occasionally, it was found that certain mutants containing a single native cysteine (and 5 serines) were unsuitable for EPR studies or were poorly expressed. In these cases, alternative mutants were constructed in which the cysteine was shifted to a neighboring site. Thus, mutants containing a single cysteine at 7 different positions (95, 116, 133, 169, 230, 265, and 347) along the length of the 40–354 IKKγ fragment were constructed, expressed and spin-labeled. The positions of the labels in the sequence are indicated in Fig. 3*E* (*solid circles*).

**FIGURE 5.**
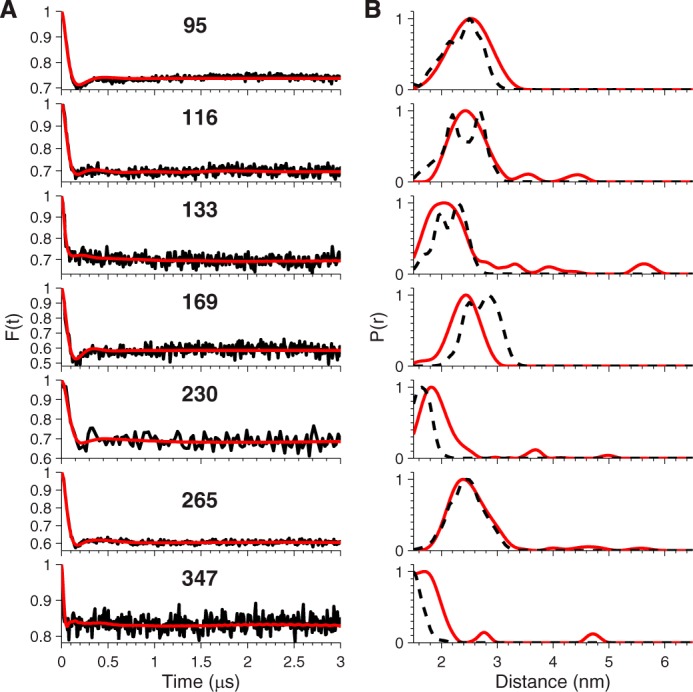
**EPR distance measurement on singly-labeled IKKγ**. *A*, background-corrected dipolar evolution (*black lines*) and their fits (*red lines*). *B*, distance distributions (*red lines*) derived from the data in *A* and predicted distance distributions (*black dashed lines*) derived from the static 3/5 hybrid model.

The background-corrected dipolar evolution and derived fits (Fig. 5*A*, *black* and *red lines*, respectively) obtained by Tikonhov regularization all show a damped oscillation that reaches a plateau after several hundred nanoseconds. The corresponding distance distributions (Fig. 5*B*, *red lines*) show a single major peak centered below 3 nm. This confirms that IKKγ is a parallel coiled-coil along its entire length consistent with the crystal structures of fragments and prediction algorithms.

##### A Static Model of IKKγ

**FIGURE 6.**
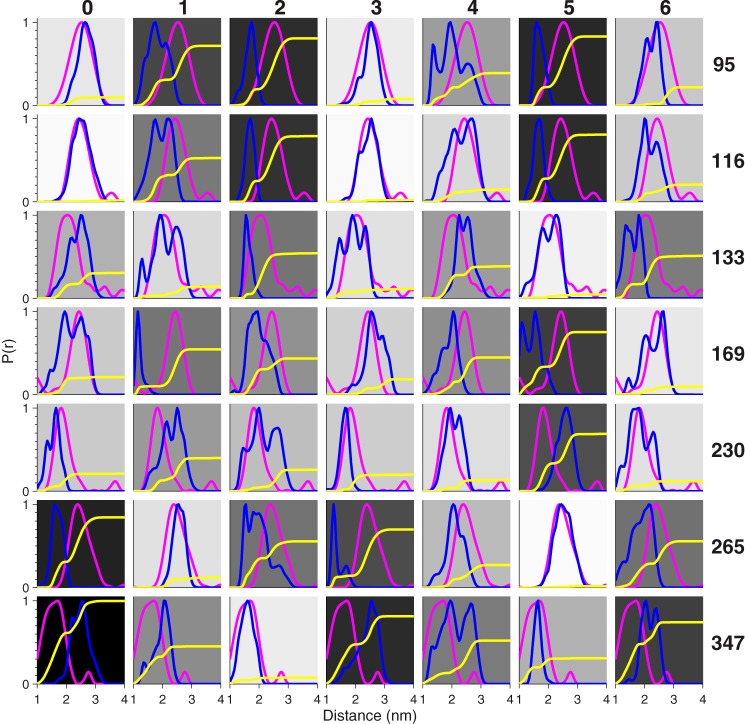
**Validation of the model structures.** Comparison of the distance distributions obtained from the EPR experiments for the singly-labeled IKKγ (*magenta lines*) with the predicted distance distributions (*blue lines*) using a rotamer library approach for the 0–6 register models. The cumulative sum of the squares of the differences between the experimental and predicted distributions is used to provide a metric for goodness of fit (*yellow lines*). The background has been *gray-shaded* according to the relative final amplitude of the *yellow lines*: a *white* background implies perfect match, whereas a *black* background implies poor agreement.

An initial model of IKKγ was constructed from a canonical coiled-coil namely Liprin B (Protein Data Bank (PDB) entry 3QH9 (30)). This was extended by iteratively overlaying carbon alpha atoms of the termini, and recording new predicted atom positions. To explore the full heptad pattern of alternative coiled-coil registers, the alignment of the sequence to the template was shifted stepwise by 0–6 residues in MODELLER (31). Distance distributions derived from the 7 models (Fig. 6, *blue lines*) using a rotamer library approach (20) were then compared with the EPR data (Fig. 6, *magenta lines*). The cumulative difference between experiment and model was used as a metric of the (dis-)agreement between them (Fig. 6, *yellow lines*). To aid visual comparison, the background of each plot has been gray-shaded according to the difference: a white implies perfect agreement; whereas black implies the largest deviation.

Register-3 and, to a slightly less extent, Register-0 gave the best agreement for positions 95, 116, 133, 169, and 230; while Register-5 gave the best agreement for positions 265 and 347. We therefore constructed two hybrid models combining the 0 or 3 and 5 registers for the N and C termini respectively, with the junction of the two registers in the region between amino acid residues Gln-231 and Lys-264. The key difference between the models is the handedness of the twist between different registers; the twist of the 0/5 hybrid is right-handed, while the twist of the 3/5 hybrid is left-handed. Both maintain the hydrophobic interface, while changing the specific angle the helices adopt relative to one another. The predicted distance distributions derived from the 3/5 hybrid gave best agreement with the single mutant EPR data (*black dashed lines*, Fig. 5*B*).

**FIGURE 7.**
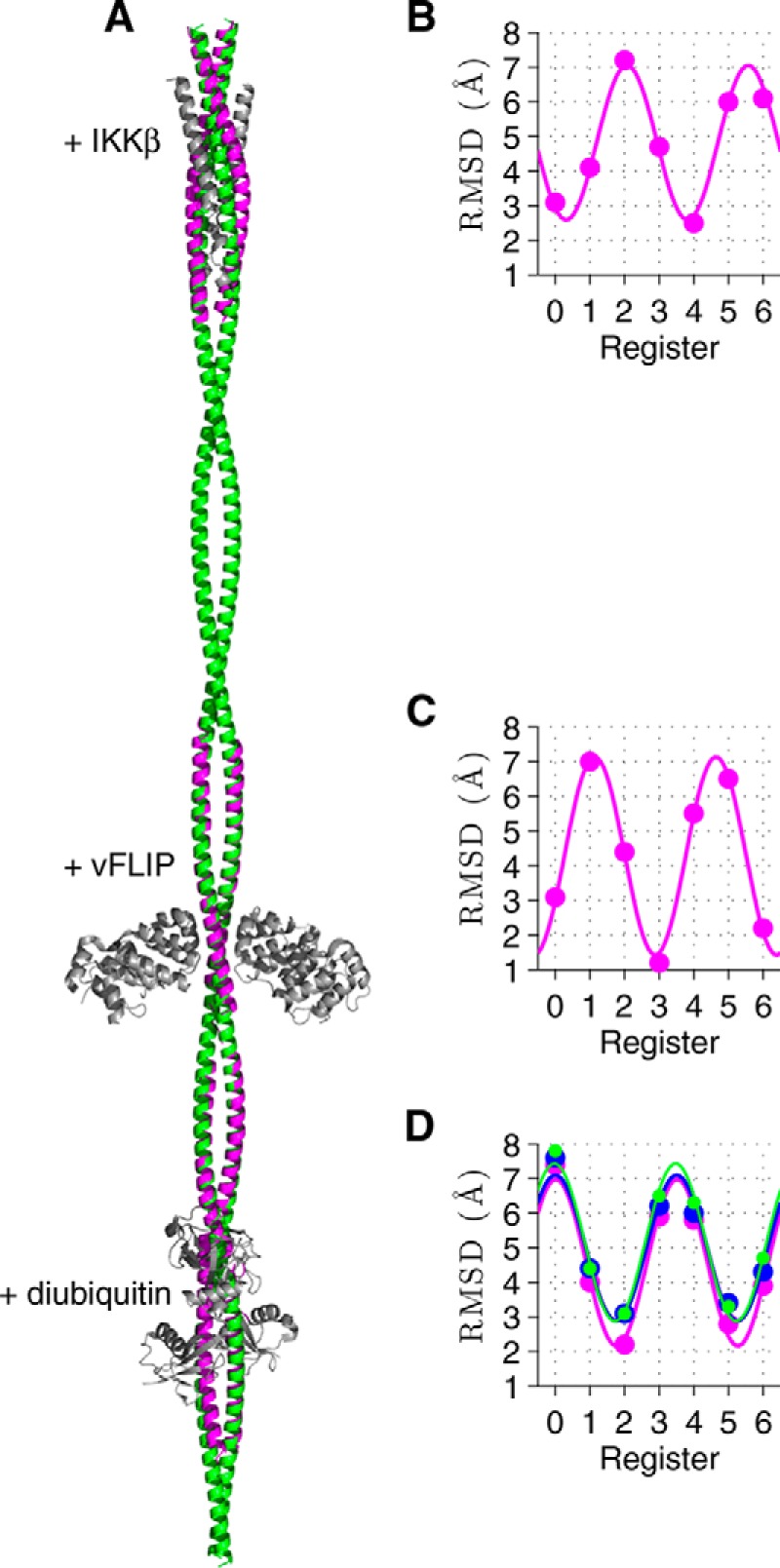
**Comparison of the 3/5 model of IKKγ with crystal structures.** *A*, 3/5 model (*green*) is overlaid with crystal structures of IKKγ fragments (*magenta*) with IKKβ, vFLIP, and diubiquitin (*gray*). Plots of the RMSDs of the positions of the non-hydrogenic atoms in the coiled-coil model structures (Registers 0–6) compared with those in the known crystal structures: (*B*) IKKγ + IKKβ (22); (*C*) IKKγ + vFLIP (12); (*D*) IKKγ ubiquitin binding region: apo (23) (*blue*), with diubiquitin (24) (*magenta*), and with Hiop (25) (*green*). The curves are fits to the RMSDs assuming a sinusoidal pattern that completes two oscillations within the heptad repeat.

An overlay of the 3/5 hybrid with three crystal structures is presented in Fig. 7*A*. To obtain a metric of the deviation of the 7 models with crystal structures of IKKγ fragments, the root mean square deviations (RMSDs) of the positions of the non-hydrogenic atoms in these regions were calculated (Fig. 7, *B–D*). As expected the RMSDs follow a sinusoidal pattern covering two complete oscillations over 7 residues. The IKKγ + IKKβ crystal structure (PDB entry 3BRT (22), residues: 50–110) has the closest match with Register-4 followed by Register-0 (Fig. 7*B*) while the crystal structure of IKKγ + vFLIP (PDB entry 3CLC (12), residues: 195–250) has closest match with Register-3 followed by Register-6 (Fig. 7*C*). By contrast, crystal structures of the ubiquitin binding region both alone (PDB entry 2ZVN (24), residues: 270–330) and in complex with diubiquitin (PDB entry 3FX0 (23)) and Hiop (PDB entry 4OWF (25), residues: 255–335) have closest matches with Register-2 followed by Register-5 (Fig. 7*D*). Thus both 0/5 and 3/5 hybrid models that we constructed from the EPR data are consistent with crystal structures giving confidence that we have derived a realistic overall view of the structure of IKKγ in solution.

##### Analysis of IKKγ Alone and in Complex with vFLIP and IKKβ using Two Pairs of Spin Labels

The results presented above do not answer the further question as to whether the coiled-coil does or does not deviate from an extended structure. Hence, to provide a more detailed survey of the IKKγ architecture and investigate any large-scale reorganization of IKKγ involving N- and C-terminal movements following association with vFLIP or IKKβ, a series of 9 cysteine double mutants (56–95; 95–133; 133–169; 158–186; 186–218; 218–250; 230–265; 265–297; and 297–331) were constructed and spin-labeled. The positions of the pairs in the sequence are given in Fig. 2*E* (*solid squares*).

**FIGURE 8.**
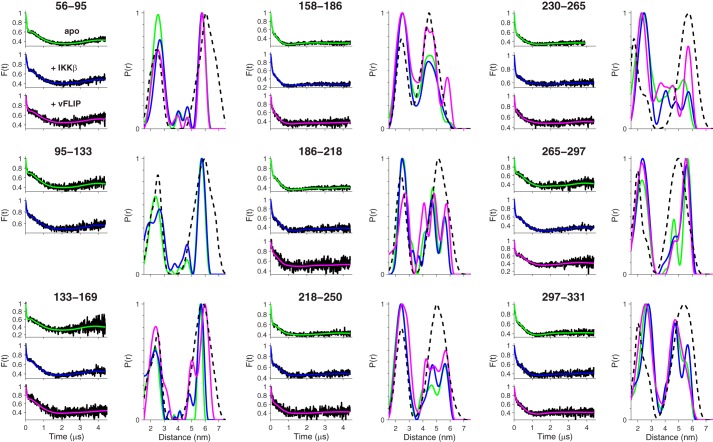
**EPR distance measurements on doubly-labeled IKKγ, apo and complexed with IKKβ and vFLIP.** Fits to the baseline-corrected dipolar evolution and corresponding distance distributions for apo IKKγ (*green lines*) and IKKγ complexed with IKKβ (*blue lines*) and vFLIP (*magenta lines*). The distance distributions for the doubly labeled IKKγ derived from the coarse-grained MD simulation of the 3/5 hybrid model are shown as a *dashed black line*.

The dipolar evolution of the 9 double mutants was recorded for IKKγ alone (green lines) and in complex with IKKβ (blue lines) and vFLIP (magenta). Inspection of the background-corrected dipolar evolution (Fig. 8) reveals that they contain both a high frequency component comparable to those seen in the spectra for pairs of spin labels (Fig. 5*A*) and a lower frequency component that extends out to several microseconds, indicative of longer distances.

Many of the data sets show small changes in profile upon binding of IKKβ or vFLIP, especially at the inflection around 200 ns, whereas the lower frequency component is largely unchanged. These observations are reflected in the corresponding distance distributions (Fig. 8). These exhibit a rich variety of shapes and all show a major peak with a maximum in the range 2–3 nm as expected for the intra-pair distance and additional peaks in the range 4–6 nm, which arise from the inter-pair distances.

56–95, 95–133, and 133–169, give single sharp long distance peaks at just below 6 nm. This indicates that the pairs of vectors joining the spin labels are close to being perpendicular and that the coiled-coil is well defined in this region. The distributions are only subtly affected by binding of IKKβ or vFLIP, although we note that vFLIP induced denaturation of the 95–133 construct for reasons that are not understood.

158–186 has a partially resolved pair of peaks centered at 4.5 nm indicating that the dihedral angle between the pairs of vectors joining the spin labels deviates from 90°. No significant change occurs when IKKβ binds, but vFLIP binding induces a broadening of the long distance distribution that may be indicative of twisting.

186–218, 218–250, and 230–265 all show a much broader distribution for the long distances, indicating that not only is the dihedral angle between the pairs of vectors joining spin labels far from 90°, but that the coiled-coil is reasonably flexible in this region. Neither IKKβ nor vFLIP have a significant effect on the distance distributions for 186–218 and 218–250, whereas for 230–265 vFLIP binding results in a remarkable sharpening of the intensity at 6 nm (magenta line), indicating that the angle between the pairs of vectors joining the spin labels tends toward 90° and that the coiled-coil has become more rigid in this complex.

265–297 and 297–331 both display a pair of long distance peaks indicating that the dihedral angle between the pairs of vectors joining the spin labels deviates significantly from 90° and that the coiled-coil is more rigid. Only minor broadening effects are observed when IKKβ or vFLIP bind.

To summarize, the double mutants provide a picture of IKKγ as an extended coiled-coil that remains largely unchanged upon binding of either IKKβ or vFLIP, although the binding of vFLIP stiffens the region spanning 230–265 (where it binds) and twists the region spanning 158–186.

##### Toward a Dynamic Model of IKKγ

The 3/5 hybrid model derived above gives reasonable agreement with the distance distributions observed with both one and two pairs of spin labels, although the predictions exhibit fine structure absent from the experimental data that is likely to have arisen because the model is static whereas IKKγ is potentially a dynamic molecule that can flex and bend. Hence, we performed a 1 μs MARTINI (17, 18) coarse-grained MD simulation of the 3/5 hybrid structure in water, with counter-ions that allowed for bending of the structure (Supplemental Movie). This dynamic model results in a better fit to the experimental distance distributions of the doubly-labeled IKKγ (Fig. 8, *black dashed lines*) supporting our conclusion that the structure is an extended coiled-coil. Additionally, visual inspection of the simulation suggests that the transition between the 3- and 5-registers that we identified as occurring in the region between Gln-231 and Lys-264 gives rise to a twist centered on residues Lys-246 to Ser-248, and that the whole structure appears to hinge about the twist.

##### Activation of IKKγ by vFLIP

**FIGURE 9.**
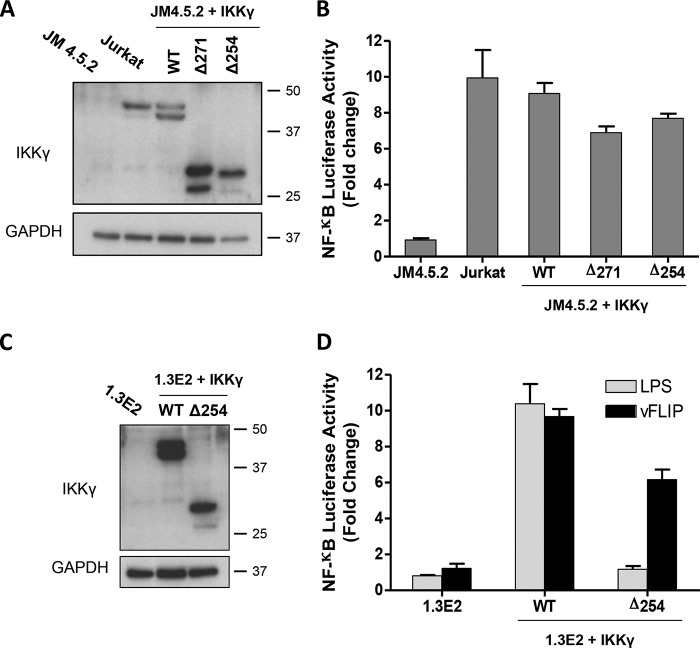
**NF-κB luciferase reporter assays for the IKKγ Δ254–419 and Δ271–419 truncation mutants and D242R mutant.** *A*, Western blot analysis of IKKγ expression level in IKKγ knock-out human T-cells JM4.5.2, parental Jurkat cells and IKKγ wild type or mutant reconstituted JM4.5.2 cells. *B*, NF-κB activation was measured using the BrightGlo Luciferase assay system 48 h after transduction with KSHV vFLIP LV (MOI = 50). Bars represent mean fold induction values ± S.D. *C*, Western blot analysis of IKKγ expression levels in IKKγ knock-out mouse PreB cell line 1.3E2, parental cells 70Z/3, and 1.3E2 cells reconstituted with IKKγ wild type; Δ254–419 mutant or IKKγ D242R. *D*, NF-κB activation was measured using the BrightGlo Luciferase assay system, 6 h after stimulation with LPS (10 μg ml^−1^) or 48 h after transduction with KSHV vFLIP LV. Bars represent mean of relative luminescence unit (RLU) values ± S.D.

We have shown that IKKγ is essentially composed of two regions with different helical registers. The C-terminal region containing the ubiquitin binding region ends just before the vFLIP binding site. To investigate whether its absence would have any impact on the capacity of vFLIP to activate the canonical NF-κB pathway, IKKγ truncation mutants were generated that terminated at Arg-254 (Δ254–419) within the discontinuity and at Glu-271 (Δ271–419) C-terminal to this region. Both mutants induced near wild type levels of activation in a NF-κB luciferase reporter assay (Fig. 9). This is in contrast to induction with LPS where they were found to be highly defective because of deletion of crucial ubiquitin binding residues C-terminal to Glu-271. Interestingly, a point mutation D242R at the vFLIP binding site within the transition between the registers renders IKKγ constitutively active in the absence of LPS (Fig. 9). Our findings thus confirm that the N-terminal region of IKKγ is alone sufficient for vFLIP-induced activation and are consistent with previous results for an IKKγ-estrogen receptor fusion protein in which IKKγ was truncated at Gly-251 (32), but also illustrate that the observed change in register is functionally important with respect to activation by endogenous cytokines.

## Discussion

It has been established that constitutive activation of the canonical NF-κB pathway by the KSHV is pivotal to viral pathogenesis having been directly linked to KS and the other lymphoproliferative disorders. Although key to this process is a physical interaction between virally encoded vFLIP and IKK modulatory subunit IKKγ that has been extensively characterized, it remains unclear how persistent activation is achieved. It has been proposed that IKKγ is held in a configuration that promotes phosphorylation of the kinases (either through autophosphorylation or recruitment of upstream kinases) distinct to that of its unbound state in response to vFLIP binding (12). This mechanism of IKK activation was first put forward for the T-cell leukemia virus oncoprotein TAX (33), a functional analogue of vFLIP.

To test this hypothesis given the absence of a full-length structure of IKKγ (either in isolation or in complex with vFLIP and IKKα/β simultaneously), we used EPR spectroscopy to obtain distances between pairs and quartets of spin-labeled cysteines positioned at intervals along the length of the IKKγ molecule. The inter-label distance distributions observed for 56–95, 95–133, 133–169, and 158–186 toward the N terminus and 265–297 and 297–331 toward the C terminus are narrow, indicating a relatively rigid structure, while those observed for 186–218, 218–250, and 230–265 in the central region of the coiled-coil are broad indicating a more flexible structure. The distance distributions were subsequently used to generate a model of the entire IKKγ molecule as well as being analyzed for changes indicative of conformational rearrangements following incubation with either vFLIP or IKKβ. Apart from stiffening of the coiled-coil observed in the vicinity of the vFLIP binding site, our results demonstrate that IKKγ does not undergo gross structural reorganization in response to binding. Furthermore, although the twisting observed for 133–169 (and the denaturation found for 95–133) indicates that subtle changes appear to be transmitted toward the N terminus of the molecule, no effect is observed for 56–96 in the vicinity of the IKKβ binding site.

The main structural insight gained is that to accommodate the change in register between the N and C termini, the region between them contains a twist located around residues Lys-246 to Ser-248. This builds tension into the structure as neither region can find a low energy conformation. As observed by x-ray crystallography, this region is essential for formation of the IKKγ·vFLIP complex (12), which in turn leads to activation of the canonical NF-κB pathway. The D242R mutation in this region appears to mimic the effect of vFLIP binding: formation of a salt-bridge to Glu-240 might induce a subtle change in structure that results in constitutive activation. The EPR data also demonstrate that binding of vFLIP stiffens this region of IKKγ.

Thus, it appears that although for the human cell, IKKγ is simply designed with different registers so that the N and C termini can perform their separate roles, namely kinase activity and regulation, respectively, the Kaposi sarcoma associated herpes virus has managed to exploit the structure of the transition between them in order to hijack the canonical NF-κB pathway.

We note that bacterial chemoreceptors, another class of coiled-coil signaling proteins have been proposed to signal through subtle modifications of a frustrated domain (34). Given the importance of this motif in signaling proteins, it is intriguing to speculate that this might be a common mechanism within the wider superfold family.

Since the induction of conformational changes can now be excluded as a potential mechanism for vFLIP-mediated NF-κB activation, alternatives need to be considered. Potentially, vFLIP may function by recruiting as yet unknown cofactors to IKKγ or through blocking those that are known to down-regulate the pathway in pro-inflammatory cytokine induced mechanisms, for example phosphatases (33). The former seems less likely considering recent reports where the absence of several factors essential to cytokine-induced activation failed to diminish vFLIP's capacity to activate the pathway (14). In agreement with this, truncation of IKKγ at Arg-254 in the region of altered helical register directly C-terminal to the vFLIP binding site has little impact on NF-κB activation. Interestingly, a crystal structure of IKKγ has recently been reported in complex with Hoip, a component of the linear ubiquitin chain assembly ligase essential for IKKγ polyubiquitination (25). The Hoip-IKKγ interface encompasses residues in close proximity to this region which may therefore have an important role in NF-κB activation by pro-inflammatory cytokines in contrast to vFLIP. Although the mechanism by which vFLIP activates the IKK complex remains unclear, given its apparent failure to induce anything but subtle conformational changes within IKKγ, we note that oligomerization of IKKβ is key to autophosphorylation (35). This may suggest that vFLIP functions to promote oligomerization of the IKKβ·IKKγ assemblies and that the D242R mutation mimics this action of vFLIP.

## Supplementary Material

Supplemental Data

## Footnotes

7 The abbreviations used are:

NF-κB: nuclear factor kappa-light-chain-enhancer of activated B cells
vFLIP: viral Flice-interacting protein
KSHV: Kaposi sarcoma-associated herpes virus
IKK: IκB kinase
NEMO: NF-κB essential modulator
EPR: electron paramagnetic resonance
MD: molecular dynamics
TNF: tumor necrosis factor
TRAF: TNF receptor-associated factors
TEV: tobacco etch virus
IPTG: isopropyl 1-thio-β-d-galactopyranoside
CVs: column volumes
LV: lentiviral vector
RMSDs: root mean square deviations.
