# Supplementary material for: Probing the Solution Structure of IκB Kinase (IKK) Subunit γ and Its Interaction with Kaposi Sarcoma-associated Herpes Virus Flice-interacting Protein and IKK Subunit β by EPR Spectroscopy
Source: J Biol Chem. 2015 May 14;290(27):16539–49. doi: 10.1074/jbc.M114.622928 (PMC4505408; doi:10.1074/jbc.M114.622928)
Supplement: Supplemental Data [file supp_290_27_16539__index.html]

Probing the Solution Structure of IκB Kinase (IKK) Subunit γ and its Interaction with Kaposi′s Sarcoma Associated Herpes Virus Flice Interacting Protein and IKK Subunit β by EPR Spectroscopy — Probing the Solution Structure of IκB Kinase (IKK) Subunit γ and Its Interaction with Kaposi Sarcoma-associated Herpes Virus Flice-interacting Protein and IKK Subunit β by EPR Spectroscopy — Solution Structure of IKKγ — Supplemental Data 

# Probing the Solution Structure of IκB Kinase (IKK) Subunit γ and Its Interaction with Kaposi Sarcoma-associated Herpes Virus Flice-interacting Protein and IKK Subunit β by EPR Spectroscopy

## Supplemental Data

- Supplemental Movie 1 (.mov, 9.1 MB) - coarse-grained molecular dynamics simulation
- Supplemental Table 1 (.xlsx, 46 KB) - List of primers
